# Chromatin reassembly following RNA polymerase II transcription

**DOI:** 10.1186/1756-8935-6-S1-O27

**Published:** 2013-03-18

**Authors:** Swaminathan Venkatesh, Michaela Smolle, Hua Li, Madelaine Gogol, Ying Zhang, Florence Laurens, Michael P Washburn, Jerry L Workman

**Affiliations:** 1 Stowers Institute for Medical Research, Kansas City, MO 64110, USA

## 

During the process of transcription elongation, the chromatin structure of transcribed sequences can be perturbed, exposing cryptic promoter-like sequences within the body of transcribed genes to function as initiation sites. Re-establishing a stable repressive structure of open reading frames requires histone chaperones, methyltransferases, deacetylases and chromatin remodeling complexes. The Set2/Rpd3S pathway is used by elongating RNA polymerase II to signal for histone deacetylation in its wake. Set2 associates with the elongating form of RNA polymerase II and co-transcriptionally methylates histone H3K36. H3K36 is recognized by the Rpd3S deacetylase complex to deacetylate histones in transcribed sequences. In recent work, we have found that a major source of co-transcriptional histone acetylation is the incorporation of soluble, Rtt109 H3K56-acetylated histones by the Asf1 histone chaperone. Set2 methylation of H3K36 promotes retention of the original histones and suppresses the incorporation of soluble histones by Asf1. By identifying factors that interact with H3K36 methylated nucleosomes, we have found that chromatin remodeling is also required to stabilize the chromatin structures of open reading frames following transcription elongation. Our studies identified the ATP-dependent chromatin remodelers Isw1 and Chd1 as two factors involved in this pathway as deletion of *ISW1* and *CHD1* enhanced the cryptic transcript phenotype caused by *set2*. Moreover, loss of *ISW1* and *CHD1* also enhanced the incorporation of new histones from the soluble pool into chromatin. Thus, retention of original histones, deacetylation of any new ones and their organization by chromatin remodeling are all required to re-establish stable chromatin resistant to cryptic transcription initiation.

